# Neighbourhood Team Development to promote resident centred approaches in nursing homes: a protocol for a multi component intervention

**DOI:** 10.1186/s12913-019-4747-0

**Published:** 2019-12-02

**Authors:** Veronique M. Boscart, Souraya Sidani, Jenny Ploeg, Sherry L. Dupuis, George Heckman, Jessica Luh Kim, Josie d’Avernas, Paul Brown

**Affiliations:** 1 CIHR/Schlegel Industrial Research Chair for Colleges in Seniors Care, Conestoga College Institute of Technology & Advanced Learning, Schlegel Centre for Advancing Seniors Care, 299 Doon Valley Drive, Kitchener, ON N2G 4M4 Canada; 2 School of Nursing, Ryerson University, 350 Victoria St, Toronto, ON M5B 2K3 Canada; 3 School of Nursing, McMaster University, 1280 Main St. W, Hamilton, ON L8S 4K1 Canada; 4 University of Waterloo, 200 University Avenue West, Waterloo, ON N2L3G1 Canada; 5 Schlegel Villages, 325 Max Becker Drive, Kitchener, ON N2E 4H5 Canada; 6 Schlegel-University of Waterloo Research Institute for Aging, 250 Laurelwood Drive, Waterloo, ON N2J 0E2 Canada

**Keywords:** Long-term care, Nursing homes, Quality of care, Care model, Culture change, Resident-centered care, Cross-functional teams, Multi-component intervention

## Abstract

**Background:**

As the demand for nursing home (NH) services increases, older adults and their families expect exceptional services. Neighbourhood Team Development (NTD) is a multi-component intervention designed to train team members (staff) in the implementation of resident-centered care in NH settings. A neighbourhood is a 32-resident home area within a NH. This paper presents the protocol used to implement and evaluate NTD. The evaluation aimed to 1) examine fidelity with which the NTD was implemented across NHs; 2) explore contextual factors associated with implementation and outcomes of the NTD; and 3) examine effects of NTD on residents, team members, family, and organizational outcomes, and the association between level of implementation fidelity and outcomes.

**Methods:**

The study employed a repeated measure, mixed method design. NTD consisted of a 30-month standardised training and implementation plan to modify the physical environment, organize delivery and services and align staff members to promote inter-professional team collaboration and enhanced resident centeredness. Training was centred in each 32-resident neighbourhood or home area. Quantitative and qualitative data were collected with reliable and valid measures over the course of 3 years from residents (clinical outcomes, quality of life, satisfaction with care, perception of person centeredness, opportunities for social engagement), families (satisfaction with care for relative, person centeredness, relationship opportunities), team members (satisfaction with job, ability to provide person centered care, team relationships) and organizations (retention, turnover, staffing, events) in 6 NHs. Mixed models were used for the analysis.

**Discussion:**

The advantages and limitations of the NTD intervention are described. The challenges in implementing and evaluating this multi-component intervention are discussed as related to the complexity of the NH environment.

**Trial registration:**

ClinicalTrials.gov ID: NCT03415217 (January 30, 2018 – Retrospectively registered).

## Contribution to the literature

- Older adults and their families expect exceptional services and care in nursing homes.
- Neighbourhood Team Development (NTD) is an approach that focuses on resident-centred care in nursing homes.
- This manuscript explores the advantages and limitations of implementing NTD, and discusses evaluation strategies to look at the fidelity of the approach and its effects on residents, team members, family and the overall organization.
- These findings contribute to the important topic of how to implement, evaluate and sustain a resident-centred model of care in nursing homes.

## Background

Population ageing is a significant global demographic trend, which will continue to increase [1]. In 1950, the number of older persons was 202 million across the world. From 1950 to 2013, this population increased four-fold, and is expected to triple by 2050 [2]. The number of older persons living with complex co-morbidities and dementia worldwide is approximately 47.5 million, with a projected increase to 75.6 million by 2030, and more than triple by 2050 [1]. Older adults with dementia often move to residential environments, such as nursing homes (NHs) to receive 24-h care to address their needs [3].

Older people residing in NHs and their families expect excellent care and services from the care team. However, literature and anecdotal evidence indicate that the quality of care in NHs is not necessarily resident and family centred, and that staff do not always collaborate as a team in providing appropriate and tailored care; instead they are task focused and work in silos, which may compromise the residents’ quality of life [4, 5]. Two main barriers that limit the ability of a care team to support residents’ care and quality of life are a) the traditional organizational NH design that does not account for resident centeredness and b) major skills misalignment so staff function independently as opposed to working as a team.

This paper describes the study protocol for implementing and evaluating *Neighbourhood Team Development* (NTD), a multi-component, organizational intervention to enhance team development and resident centeredness in NHs. The NTD training program was informed by the work of Eden Alternative, and was originally developed by Vivage Quality Health Partners**©** [6] and others [7, 8] examining care teams and resident centeredness in NHs. In 2010, the Eden Alternative designed a training course for Neighbourhood Guides, staff in NHs who are to support and mentor teams to implement resident-centred care at the “neighbourhood” level (a limited number of rooms and living space within the larger NH). Although the course offered a strong foundation in team development, it lacked clearly defined integration of resident centred practices, was not aligned with the organizational context in which neighbourhoods were to be implemented in the organization interested in adopting the program, and did not provide guidance on how the program implementation should be evaluated.

Schlegel Villages, a large group of private retirement and NHs in Ontario, Canada, and the investigators used this course (with permission) as a starting point to develop an integrated change program, NTD, that extends the training of individual neighbourhood guides within a large organizational change process. NTD was implemented over a three-year period, involving standardised training followed by care team training (per neighbourhood).

NTD consists of three components: 1. Modifying the physical NH environment, 2. Reorganizing the organization and delivery of care and services, and 3. Aligning team members to collaborate in providing resident-centred care. In addition, NTD intentionally includes strategies to address traditional organizational design and skills misalignment. This focus makes NTD unique in that no other resident centred programs have integrated these elements. Core beliefs of NTD include consistent team assignments to a dedicated neighbourhood, respect of residents’ choice and autonomy, and self-directed work teams on each neighbourhood (Table 1).

**Table 1:** Core Beliefs of NTD

Core Belief	Description
Consistent team assignments to dedicated neighbourhood	• Redesign of organizational structure to move from siloed departments and hierarchical management to cross-functional teams. • Empowering cross-functional neighbourhood teams to be more effective and responsive to resident-centred decision-making. • Cross-training and blending of roles to allow for greater flexibility and cohesiveness of care by eliminating task orientation and job delineation.
Respect for residents’ choice and autonomy	• Redesign of NH services to fit needs and expectations of residents with complex needs. • Redesign of physical environment to promote resident-centeredness.
Self-directed work teams per neighbourhood	• Financial framework of accountability by emphasizing team members working to full scope of practice while providing better coordinated care. • Solidified approach to seamless team functioning where residents come first.

There is a steadfast growth in NHs adopting resident centred practices. To be resident-centred is to have a home as an environment that directs and supports close relationships among team members, residents and their families, residents’ choices and team member empowerment and collaborative decision making, with the goal of improving quality processes.

Public pressure on NHs and the potential for decreased care quality for complex residents within a traditional design encourages NH leaders and practitioners to start changing their care models. Given this trend, there is a real need to identify effective resident-centred care models and build evidence informed implementation and evaluation plans. The complexity of care in NHs calls for a multi-pronged approach to implementation and evaluation and is likely the reason why several narrowly focused interventions were neither successful nor sustainable.

Researchers, professionals and policy makers agree that the quality of care delivered in NHs is often inconsistent and generally suboptimal. Empirical evidence [3] demonstrates several interconnected concerns including increased resident care needs, low staff-resident ratios and limited team work [8], regulatory standards that are inconsistent with resident-centred care [9], resident and family dissatisfaction [10], staff burnout and low retention [11, 12], over- regulation, [13], lack of innovative leadership [14], and concerns about current and future capacity to respond to the needs of those with ADRD [15].

Overall, a review of studies that evaluated resident centred care models showed promising results related to the residents’ quality of life and resident-centred care. Yet, findings for team members, family and organizations are limited, with at times mixed results on satisfaction and turnover data. This might be due to the limited understanding and implementation of resident-centred interventions. There remains little data on sustainability of programs, as well as effectiveness of program components.

### Aims

The goal of this current study was to implement and evaluate NTD. The specific objectives were to: 1. Examine the fidelity of the program when implemented in a multi-home NH organization; 2. Explore contextual factors associated with implementation and outcomes; 3. Examine the effects of the program on residents, team members, family, and organizational outcomes and the association between the level of implementation fidelity and outcomes, and; 4. Determine the mechanisms through which NTD exerts its effects.

## Methods

### Design

A multi-component intervention for a single group was used. NTD involved a 30-month implementation plan, starting with a 3-day training for all management and neighbourhood coordinators (NCs), team leaders who are trained to coordinate care service and delivery for two specific neighbourhoods and is the designated primary contact for residents and their family members, followed by individual team development on all neighbourhoods across the home for 12 months. Upon the start of the 2nd implementation year, the second training session for management and NCs took place (3-day training), again followed by a 12-month implementation plan on the neighbourhoods. A third and last training with management and NCs took place at the start of the third implementation year, followed by a 6-month implementation.

Training for management and NCs was delivered by NTD experts over the course of two consecutive days, twice per year, followed a training manual, and included competencies in care coordination, care and service delivery for the neighbourhoods, team’s interpersonal skills and care competencies, care decision support based on group processes, awareness and knowledge to promote residents’ choices, and skills for ongoing supervision and stewardship of the team. Individual team development per neighbourhood was delivered by the NCs on a quarterly base (4 h per session) and consisted of interactive group modules building capacity in team attributes, professional competencies relating to knowledge and skills to support resident centeredness (i.e., resident-centred decision making, reprioritizing care consistent with the residents’ preferences) and competencies in relational care and in the delivery of care and services. The researchers adhered to the Standard Protocol Items: Recommendations for Interventional Trials (SPIRIT) guidelines.

### Sample and recruitment

This study aimed for a rigorous evaluation of the process and complex outcomes of NTD implementation and used a pragmatic real-world effectiveness approach; as such, no formal eligibility criteria or sample size calculations were completed. Data were available from 11 LTC homes, serving 192 residents, which is approximately 2112 residents in total. The number of team members providing direct care varied over time and by LTC home. The sample for the study included residents (*n* = 1110), family members (*n* = 62) and team members (*N* = 2173) of 11 NHs in Ontario, Canada. Given data availability, the power of this study to detect an increase in residents’ self-reported quality of life was based on a one-sample t-test. Assuming an effect size of 0.2, standard deviation of 10, and sample size of 2112, the study will have a power of 0.81 to test change over time. Sample size calculations were performed using SAS University Edition (SAS Institute Inc., North Carolina, USA).

### Research team

In order to gauge the progress of the study, the NTD research team met monthly to review implementation and evaluation procedures. During the course of the study, the overall research team met quarterly to discuss analysis and results of the study.

### Data collection

Processes and outcomes of NTD include data gathered from residents, families, care teams, and the organization (Table 2). Measures for the residents included quality of life, quality of life for people with ADRD, enhanced care experiences, resident centredness and choice and control, satisfaction with NTD*,* and functional and cognitive status indicators (Selected items from the Minimum Data Set –LTC 2.0) (Table 3). Family measures included proxy quality of life indicators, enhanced care experiences, involvement in care and decision making, and satisfaction with NTD. Measures for the team included beliefs and actions, perception of role enactment and team functioning, continuity in assignments, enhanced care experiences, staff-resident relationships and job satisfaction. Organizational descriptors included work distribution, staff turnover and retention. Data were collected through observations, interviews, focus groups, surveys, medical record and chart reviews, and database reviews. Data were collected between March 2013 and March 2017. Data analysis and manuscript writing is ongoing.

**Table 2:** Variables and Measurement Schedule

Sample	Category	Variables	Measurement Schedule
Resident	Demographics	Age, gender, marital status, unit	All assessments
	Quality of life indicators	Quality of Life Scales for Nursing Home Residents by Kane	Baseline and every 6 months
	Quality of life for people with ADRD	Observing Quality of Life in Dementia (OQOLD) Measure	baseline and every 6 months
	Resident centredness	CARE Profile	baseline and every 6 months
	Resident’s choice and control	Health-Related Quality of Life Measure	Baseline and every 6 months
	Functional and cognitive status	Depression Rating Scale, Index of Social Engagement, weight, CHESS scale, feeding tube, VAS Pain Scale, pressure ulcers (BWAT), physical restraint use, Activities of Daily Living (ADL Self-Performance Hierarchy Scale), indwelling catheter use, dlirium symptoms (CAM), number of falls, UTIs, new infections, use of anti- psychotics and anti- depressants, number of acute care admissions	Baseline and every 6 months
	Semi-structured interviews	Experiences related to the NTD program implementation	Every 12 months
Staff	Demographics	Position, proportion of time spent with residents, employment status (i.e. full-time, part-time, casual), shift type (i.e. day, evening, night), education, years worked in NH, years worked at current NH

**Table 3:** Measurement Tools

Scale	Scores	Reference
Cognitive Performance Scale (CPS)	The CPS showed substantial agreement with the MMSE in the identification of cognitive impairment; the sensitivity was .94 (95% confidence interval [CI]: .90, .98), the specificity was .94 (95% CI: .87, .96), and the diagnostic accuracy as measured by the area under the receiver operating characteristics (ROC) curve was .96 (95% CI: .88, 1.0).	Hartmaier, S.L., Sloane, P.D., Guess, H.A., Koch, G.G., Mitchell, C.M., & Phillips, C.D. (1995). Validation of the minimum data set cognitive performance scale: Agreement with the mini-mental state examination. *Journals of Gerontology Series A: Biological Science and Medical Science, 50*, M128–33.
Depression Rating Scale (DRS)	Correlations of 0.69 with Cornell scale and 0.70 with Hamilton Depression Rating Scale. More sensitive and specific than 15-item Geriatric Depression Scale (AUC > 0.80).	Burrows, A.B., Morris, J.N., Simon, S.E., Hirdes, J.P., & Phillips, C. (2000). Development of a minimum data set-based depression rating scale for use in nursing homes. *Age and Ageing, 29,* 165–72.
Activities of Daily Living (ADL)-long form	Interrater reliability > 0.75, internal consistency is high (Chronbach’s alpha = 0.954).	Morris, J.N., Fries, B.E., & Morris, S.A. (1999). Scaling ADLs within the MDS. *Journals of Gerontology Series A: Biological Science and Medical Science, 54* (11), M546–53.
Activities of Daily Living (ADL) –short form	Interrater reliability > 0.75, internal consistency is high (Chronbach’s alpha = 0.90).	Morris, J.N., Fries, B.E., & Morris, S.A. (1999). Scaling ADLs within the MDS. *Journals of Gerontology Series A: Biological Science and Medical Science, 54* (11), M546–53.
Index of Social Engagement (ISE)	Internal consistency is moderate (Chronbach’s alpha = 0.79).	Mor, V., Branco, K., Fleishman, J., Hawes, C., Phillips, C., Morris, J., & Fries, B. (1995). The structure of social engagement among nursing home residents. *Journals of Gerontology Series B: Psychological Science and Social Science, 50* (1), P1–8.
Pain Scale	Kappa between VAS and Pain was 0.707; variance explained 56%.	Fries, B.E., Simon, S.E., Morris, J.N., Flodstrom, C., & Bookstein, F.L. (2001). Pain in

### Data analysis

The evaluation of NTD consists of analysing findings, which aim to address four main objectives:

#### Objective 1: examining the Fidelity of the program

This objective aims at measuring if NTD is delivered as designed. This is addressed by conducting descriptive analyses of the structured observations of staff-resident and team interactions; the surveys and questionnaires; the description of job enactment; and the resident, team members and family satisfaction surveys (Table 2). The quantitative data on fidelity of the program implementation and the observational data will be analyzed descriptively for each site to determine the extent of variation from the planned implementation. In addition, differences in fidelity will be examined across participating sites, using mixed methods. Qualitative data from the semi-structured interviews will be undergoing a constant comparative analysis and will be reviewed separately by two investigators using NVivo 10. Thematic content emerging from the transcripts using an inductive coding technique will be subsequently organized into categorized concepts. The thematic frameworks emerging from the interviews will assist in understanding factors that may contribute to variation in program implementation across the neighbourhoods**.**

#### Objective 2: exploring contextual factors associated with implementation and outcomes

This objective aims to determine if and how contextual factors might have modified NTD effectiveness. A descriptive analysis of the contextual factors of NTD implementation and outcomes will be conducted: including number and training of NCs, changes in staffing, knowledge and skills required for implementation, physical changes to care environment, and any other events during implementation. Findings of the descriptive analysis will be site specific first. Further investigation of how the NTD model effects were modified by the specific context will be carried out using mixed methods. A qualitative analysis will help in uncovering key contextual factors associated with the implementation and the outcomes and will help to explore emerging themes and patterns in more detail.

#### Objective 3: examine effects of the program on residents, team members, family, and organizational outcomes, and the association between the level of implementation Fidelity and outcomes

The effect of NTD on outcomes will be measured by conducting a descriptive analysis (including mean, standard deviation and frequencies) of all outcome indicators at each data collection point. Mixed models will be conducted on the quantitative data to examine changes in and resident’, family’, and team member’s outcomes variables over time and between groups, while accounting for possible site differences. Qualitative data from the semi-structured interviews will be undergoing a constant comparative analysis. Organizational data will be analyzed by conducting a descriptive analysis on job descriptions and work distribution, skill mix, supportive organizational systems, and staff turnover and retention. Effect of NTD on residents, team members, family, and organization will be described in the context of program.

#### Objective 4: determining the mechanisms through which NTD exerts its effects

It is hypothesized that the implementation of NTD will lead to improved knowledge, skills, competencies in relation to resident-centredness and collaborative work among team members which in turn should lead to enhanced neighbourhood team practices relative to meeting the residents’ needs (in all domains), resulting in enhanced resident outcomes. A latent growth curve analysis model (combining multi-level modeling and structural equation modeling) will be applied to determine the mechanisms through which NTD exerted change(s) on the outcomes per neighbourhood and for the combined data. As well, a constant comparative analysis of all data will be conducted to uncover any underlying mechanisms that are not explained by the descriptive approaches. Findings from this objective combined with findings from the first three objectives will provide insight in fidelity, contextual factors, effectiveness, and the mechanisms of the effectiveness of NTD on team cohesiveness and resident-centred care in NHs.

### Ethical considerations

This study received ethics clearance by the Research Ethics Board at the agency (REB-118). Informed consent was obtained from residents, family members and staff. Information letters, group and individual information sessions to explain the study were provided to residents, family members and staff. Investigators and research assistants met with participants individually to obtain written and verbal consent.

## Discussion

This study brings an innovative intervention, NTD, to enhance resident centred care in NHs, consistent with the urgent priorities highlighted in previous research, that is, the redesign of NH services to fit the need and expectations of NH residents and addressing the spectrum of care for older persons with complex needs involving dementia [3, 5]. This study aims to provide evidence that NTD can enhance NH care, including preserving residents’ autonomy and dignity, emphasizing quality of life, promoting best practices, all while working within a framework of accountability by emphasizing team members working to their full scope of practice while providing better coordinated care. This multi-component, mixed method study has the potential to generate high impact results and demonstrate the use of tools and methodologies for NHs and dementia care and research. Furthermore, research goals include a new NH model, better care experiences for residents and families, a solidified approach to seamless team functioning, and a program in which every member is used to its fullest capacity and where the residents come first.

Although NTD could be perceived as an easy potential solution for better quality outcomes in NHs, it is important to realize that a large organizational change, such as the one required for NTD implementation, requires significant organizational commitment and leadership. Financial contributions are required for the physical (re) designs, NTD implementation, and the gathering of the data. The authors’ experience with in-kind and financial support and a strong commitment by the organization’s leadership and teams has led to successful recruitment, implementation and data collection. In addition, this study provides an opportunity to test the data collection measures and procedures, software to extract required data from existing data bases, survey completion sessions, site specific and detailed electronic databases, and electronic data collection logs to keep track of the large amount of data. This information allows the research team to fine tune the outcome measures, the research staffing and allocation, and the budget.

Lastly, the authors observed that previous studies or practices aiming to implement RCC models have been curtailed due to the lack of available funds to cover wage/replacement cost for the participation of NH teams. As for any organizational and care model change, one’s biggest investment ought to be in its team member in order to have quality outcomes. For NTD implementation, all participant compensation costs are covered by the organization, in addition to meeting rooms for the training and neighbourhood team sessions.

## Conclusions

This study is the first in a program of research that will lead to a different way of organizing NHs, innovations in how teams collaborate to best serve the needs of older persons, and important insights on what factors need to be in place for sustainable RCC models. Future research will focus on a multi-site, controlled study to further evaluate the effectiveness of NTD on residents’ well-being. This study is one step forward toward resident centred care delivery in a competent and compassionate manner for people in NHs.

## Data Availability

The data that support the findings of this study are available from the participating LTC homes, but restrictions apply to the availability of these data, which were used under license for the current study, and so are not publicly available. Data are however available from the authors upon reasonable request and with permission of the participating LTC homes.

## Abbreviations

NH: Nursing Homes
NTD: Neighbourhood Team Development
